# Prevalence and resistance patterns of commensal *S. aureus* in community-dwelling GP patients and socio-demographic associations. A cross-sectional study in the framework of the APRES-project in Austria

**DOI:** 10.1186/s12879-015-0949-1

**Published:** 2015-05-16

**Authors:** Kathryn Hoffmann, Casper D. J. den Heijer, Aaron George, Petra Apfalter, Manfred Maier

**Affiliations:** Department of General Practice and Family Medicine, Centre for Public Health, Medical University of Vienna, Kinderspitalgasse 15/1st floor, 1090 Vienna, Austria; Department of Medical Microbiology, Maastricht University Medical Centre/CAPHRI, Maastricht, The Netherlands; Department of Community and Family Medicine, Duke Medical Center, Durham, NC USA; Institute for Hygiene, Microbiology and Tropical Medicine (IHMT), National Reference Centre for Nosocomial Infections and Antimicrobial Resistance, Elisabethinen Hospital Linz, Linz, Austria

**Keywords:** Staphylococcus aureus, MRSA, Commensal antibiotic resistances, Primary health care, Austria

## Abstract

**Background:**

The aim of the present study was to assess the prevalence and resistance of commensal S*. aureus* in the nasal microbiota of community-dwelling persons in Austria, as well as to identify possible associations with socio-demographic factors. Multi-drug resistance in this population was additionally studied.

**Method:**

This cross-sectional study was conducted within the context of the European APRES project. In nine European countries, nasal swabs were collected from 32,206 general practice patients who received care for non-infectious reasons. In Austria, 20 GPs attempted to recruit 200 consecutive patients without infectious diseases, with each patient completing demographic questionnaires as well as providing a nose swab sample. Isolation, identification, and resistance testing of *S. aureus* were performed. Statistical analyses included subgroup analyses and logistic regression models.

**Results:**

3309 nose swabs and corresponding questionnaires from Austrian subjects were analyzed. *S. aureus* was identified in 16.6 % (n = 549) of nose swabs, of which 70.1 % were resistant against one or more antibiotics, mainly penicillin. *S. aureus* carrier status was significantly associated with male sex (OR 1.6; 1.3–2.0), younger age (OR 1.3; 1.0–1.8), living in a rural area (OR 1.4; 1.1–1.7) and working in the healthcare sector (OR 1.5; 1.0–2.1). Multi-drug resistances were identified in 13.7 % (n = 75) of the *S. aureus* carriers and 1.5 % (n = 8) tested positive for MRSA. The highest resistance rate was observed against penicillin (64.8 %), followed by azithromycin (13.5 %) and erythromycin with 13.3 %.

**Conclusion:**

This study describes the prevalence and resistance patterns of commensal *S. aureus* in community-dwelling persons in Austria and shows that differences exist between socio-demographic groups. Demographic associations have been found for *S. aureus* carriers but not for carriers of resistant *S. aureus* strains. Only two thirds of *S. aureus* strains were found to be resistant against small spectrum penicillin. As it is recognized that one of the corner stones for the containment of antibiotic resistance is the appropriate prescription of antibiotics in the outpatient sector, this finding lends support to the avoidance of prescription of broad-spectrum antibiotics to treat *S. aureus* infections in the community.

## Background

One of the major challenges for healthcare systems around the world is the growing prevalence of antibiotic resistance (AR) [1]. The World Health Organization’s 2014 report on AR began with an introductory statement that raised concern for, *“A post-antibiotic era—in which common infections and minor injuries can kill—far from being an apocalyptic fantasy, is […] a very real possibility for the 21st century.*[1]*”* AR is known to be associated with an increased morbidity and mortality [1–6]. At least four reasons for recent increasing rates in AR have been suggested: 1) the excessive use of antibiotics by humans, including antibiotic overtreatment fostered by over-the-counter selling of antibiotics and inappropriate prescriptions of antibiotics for viral infections [1, 7–10], 2) influences of travelling [11], 3) the excessive use of antibiotics in livestock breeding [1, 12–15], and 4) the stagnancy in development of new generations of antibiotics [1, 16]. Together, this has led to a large output of resistant bacteria into the environment, where resistance genes can disseminate easily [1, 17–19]. These factors for AR imply that community exposures and ambulatory care have a large impact on acquisition and resistance. However, the majority of existing information on antibiotic resistance pattern has been obtained from hospitalized patients, rather than from samples of community-dwelling persons [9, 20, 21]. Given that community-acquired AR differ from patterns observed in hospitalized patients, identifying the prevalence and resistance in the general population would provide an important source of knowledge on the acquisition of resistance in pathogens [1].

While *S. aureus* is a common part of the normal human microbiota, it is also often found to be responsible for several severe infections [22–24]. *S. aureus* is of particular interest due to its special capacity to acquire resistance, as exemplified by the recent steep increase in AR levels of *S. aureus* strains against penicillin [25]. Furthermore, circumstances such as the increasing prevalence of methicillin-resistant *S. aureus* (MRSA) that result in limited treatment options, continue to press the growing international concern and need for surveillance of the situation in the non-hospitalized setting [1, 26, 27].

In the context of the EU-project “APRES – the appropriateness of prescribing antibiotics in primary health care in Europe with respect to antibiotic resistance” [28], it was our aim to gain detailed information about the prevalence and resistance of commensal *S. aureus* of community-dwelling GP patients in Austria. Specifically, we aimed to evaluate a community population for nasal *S. aureus* carriage and to observe any associations with socio-demographic data, as well as to assess for multi-drug resistance. Moreover, we performed subgroup-analyses on those patients carrying *S. aureus* with resistance to any antibiotic aside from penicillin, as this group may be associated with higher risks for acquiring AR and multi-drug resistances.

## Methods

### Design

This cross-sectional study took place in the context of the European APRES project, which included nine European countries and a methodology that has been described in depth in several publications [8, 28–30]. Therefore, we describe in this publication only the methods relevant for Austria. [22] The study analysis was designed in accordance with the STROBE statement for cross-sectional studies [31].

#### Recruitment of study participants in Austria

First, twenty general practitioners (GPs) were recruited via electronic invitation by the Austrian Society of General Practice and the research network of the Department of General Practice at the Medical University of Vienna. In line with the APRES project, those recruited constituted a fair representation of the national GP population with regard to sex, age and federal state [32]. Additionally, GP demographics were included in this study. Between November 2010 and July 2011 these twenty GPs were asked to identify 200 consecutive patients aged four years and older. Detailed inclusion and exclusion criteria for the patients were published elsewhere [28, 30]. Most important for this analysis was the exclusion criterion that the patients’ consultation had to be due to a non-infectious disease and that the patients did not take antibiotics for a minimum of three months prior to the study. Additional important exclusion criteria included those living in a nursing home, those with any hospitalization in the three month period prior to the study, the immunocompromised, and all those aged less than 4 years. A nose swab was collected and each patient then completed two questionnaires regarding their socio-demographic data. Among this was the APRES patients’ questionnaire and an additional questionnaire designed by the Department of General Practice at the Medical University of Vienna, with further questions relating to demographics. The additional questionnaire is described in detail in the publication of Hoffmann et al. [30]. For those patients between four and 14 years, a parent completed the questionnaires to ensure the reliability of the data.

#### Material

The nose swabs were sent within 24 hours to the national laboratory (Institute of Hygiene, Microbiology and Tropical Medicine, Elisabethinen Hospital, Linz, Austria) for the identification and isolation of *S. aureus.* Charcoal swabs were used as transport medium (Transystem, 114 C; Copan Italia, Brescia, Italy). The swabs were sent to the laboratory using special envelopes to ensure security and fast delivery of the swabs. All laboratories used standardized protocols for the identification of *S. aureus* [28]. Those swabs that were found to have isolated *S. aureus* strains were sent to the APRES central laboratory for further AR testing. All AR testing was performed in accordance with the European Committee on Antimicrobial Susceptibility Testing (EUCAST) guidelines and EUCAST epidemiological cut-offs were used as breakpoints [33]. Antimicrobial susceptibility testing was performed for multiple antibiotics, including, azithromycin (AZITH), ciprofloxacin (CIP), clindamycin (CLIND), daptomycin (DAPT), erythromycin (ERY), gentamicin (GENTA), linezolid (LIN), oxacillin (OXA), penicillin (PEN), tetracyclin (TETRA), trimethoprim-sulfamethoxazole (TRISUL), and vancomycin (VANC). *S. aureus* ATCC 29213 was used as control strain. All isolates susceptible to CLIN and resistant to ERY were tested for inducible CLIIN resistance by means of the D-test [34]. In the case of a positive D-test, strains were considered resistant to CLIN. Additionally, the presence of MRSA strains was evaluated. The MRSA resistance screening was previously described in detail by den Heijer et al [8].

Multi-drug resistance was defined as a *S. aureus* strain that was resistant to three or more antibiotics.

#### Patients’ information

Patient information in the two questionnaires used for this study included sex, age, educational level, country of origin, location of residence, and profession. Age was clustered into four groups: age 4-18 years (children), 19–39 years (young adults), 40–64 years (older adults), and 65 years and older (elderly). The highest educational level attained was surveyed in three groups: primary, secondary and tertiary education. Tertiary education was defined as completion of university or any further or post graduate education. Country of origin was assessed with the question “What is your country of origin?” This variable was grouped into four clusters: 1) Austria, 2) European Union (EU) 15 countries including European Free Trade Association (EFTA) countries (EU15+), 3) new EU 28 countries (EU28), and 4) all other countries. Location of residence was dichotomized into urban areas (big and intermediate cities) and rural areas (small cities, villages and countryside). Classification of the area was applied by using the European DEGURBA (degree of urbanization) classification [35]. Profession was assessed by “Do you work in any of the following occupational fields?” with the answer categories “healthcare”, “livestock farming”, “kindergarten teacher/ (day) nanny”, or “others”. All socio-demographics were defined as independent variables.

#### Data analyses

The initial analysis was carried out for the distribution of socio-demographic data for all patients, *S. aureus* carriers, *S. aureus* carriers with antibiotic resistance to any antibiotic as well as any antibiotic besides PEN, and those carriers of MRSA. This was performed using descriptive statistical methods (absolute and relative frequencies and cross-tabs) and the Chi-Square Independency or Fisher’s Exact test for categorical variables. If independency could not be proven, the two-proportion z-test to compare two or more column proportions including the Bonferroni method for multiple testing was used to determine which particular categories were not independent. Next, multivariable logistic regression models were conducted [36]. In the first regression model, *S. aureus* carrier status was the dependent variable, while those *S. aureus* carriers with AR were recognized as the dependent variable for the second regression model and those with AR besides PEN for the third model. All socio-demographic variables were included simultaneously in the model and a backward regression model was performed. Variables were excluded at a level of 0.20. We chose a *p*-value cut-off point of 0.2 for the backward selection as more traditional levels, such as 0.05, can fail to identify variables known to be important [37, 38]. This was followed by purposeful testing of eventual missed effects of the excluded variables. This step can be helpful in identifying variables that, by themselves, are not significantly related to the outcome, yet make an important contribution in the presence of other variables. At the end of this final step, the preliminary main effects model could be analyzed [37]. Additionally, the regression models were adjusted for GP practice code to account for any possible inter-practice effect. This was performed by building a dichotomous dummy variable for each GP practice, which was included in the models.

Then, the number of resistances of *S. aureus* against AB was assessed and the distribution of the socio-demographic variables in relation to the number of antibiotics resistant to *S. aureus* calculated by means of descriptive statistical methods.

The significance level for all calculations was p < 0.05 and the confidence interval was 95 %. SPSS Statistics version 22.0 was used for the statistical analyses.

### Ethical considerations

The study was approved by the Ethics Committee of the Medical University of Vienna (EC # 568/2010).

Each participant had to complete a written informed consent form prior to participation. If the patient was younger than 18 years, both a parent and the child each completed written informed consent forms. Those aged 4 to 13 completed a special consent form for children, while those aged 14 to 18 completed a form for adolescents.

## Results

Altogether, 3380 nose swabs of GP patients were collected in Austria. Of those, 3309 were eligible for this analysis because patients met the inclusion criteria and had complete *S. aureus* and questionnaire data. The distribution of the socio-demographic data for the whole sample is described in Table 1. *S. aureus* was identified in 16.6 % (n = 549) of patients’ nasal swabs, of which 70.1 % (n = 385) of the *S. aureus* found were resistant to one or more antibiotics. MRSA was identified in 1.5 % (n = 8) of *S. aureus* carriers (8). The highest resistances were observed to PEN with 64.8 % (n = 356), followed by AZITH with 13.5 % (n = 74), ERY 13.3 % (n = 73), CLIND 11.1 % (n = 61), TETRA 3.5 % (n = 19), GENTA 2.2 % (n = 12), CIP 1.5 % (n = 8), OXA 1.5 % (n = 8), and TRISUL with 0.2 % (n = 1).

**Table 1:** Distribution of the socio-demographic variables among *S. aureus* carriers, carriers with resistances and MRSA carriers

Variable	Sub-variable	All	*S. aureus* carrier	*S. aureus* carrier with resistances	*S. aureus* carrier with resistances (besides PEN)	MRSA carrier
		% (n)	% (n)	% (n)	% (n)	% (n)
All		100 (3309)	16.6 (549)	70.1 (385)	64.8 (356)	1.5 (8)
Sex	Female	56.6 (1862)_a_	13.9 (257)_a_	72.8 (187)	66.1 (170)a	2.3 (6)
	Male	43.6 (1428)_b_	20.3 (288)_b_	68.4 (197)	64.2 (185)a	0.7 (2)
p		<0.001	<0.001	0.235	0.640	0.234
Age	4–18	4.2 (139)	20.1 (28)_a_	71.4 (20)	67.9 (19)	0
	19–39	27.6 (913)_a_	18.2 (166)_a,b_	74.1 (123)	71.1 (118)	2.4 (4)
	40–64	45.1 (1493)_b_	16.8 (251)_a,b_	68.5 (172)	62.2 (156)	1.2 (3)
	65+	23.1 (764)_c_	13.6 (251)_b_	67.3 (70)	60.6 (63)	1.0 (1)
p		<0.001	0.043	0.580	0.206	0.651
Educational level	Primary	49.0 (1580)_a_	16.9 (265)	69.4 (184)	65.3 (173)	0.4 (1)
	Secondary	37.3 (1203)_b_	16.2 (194)	69.1 (134)	62.9 (120)	1.5 (3)
	Tertiary	13.7 (442)_c_	16.8 (74)	70.3 (52)	64.9 (48)	5.4 (4)
p		<0.001	0.891	0.991	0.741	0.018
Country of origin						
	Austria	86.0 (2806)_a_	16.7 (467)	69.0 (322)	63.6 (297)	1.5 (7)
	EU 15+	3.2 (103)_b_	21.6 (22)	77.3 (17)	72.7 (16)	0
	New EU 28	2.8 (94)_b_	18.1 (17)	76.5 (13)	70.6 (12)	5.9 (1)
	Others	7.9 (259)_b_	14.0 (36)	75.0 (27)	69.4 (25)	0
p		<0.001	0.352	0.742	0.688	0.698
Location of residence						
	Urban	43.9 (1461)_a_	13.8 (200)_a_	67.2 (135)	59.2(119)_a_	1.5 (3)
	Rural	56.1 (1867)_b_	18.8 (345)_b_	71.8 (250)	68.1 (237)_b_	1.4 (5)
p		<0.001	<0.001	0.287	0.035	0.828
Job						
	Health care	5.8 (194)_a,b_	21.6 (42)	71.4 (30)	64.3 (27)	7.1 (3)
	Livestock farming	2.9 (96)_b_	16.7 (16)	62.5 (10)	56.3 (9)	0
	Kindergarten teacher/nanny	1.9 (62)_b_	11.3 (7)	85.7 (6)	85.7 (6)	0
p	Others	78.8 (2621)_c_	16.5 (431)	70.3 (303)	64.7 (279)	0.9 (4)
	Not known	10.7 (355)_a,b_	15.7 (53)	67.9 (36)	66.0 (35)	1.9 (1)
		<0.001	0.302	0.866	0.755	0.119

^a,b,^The subscript letters represent a subset of the variable category which is not significantly different at a significance level of p < 0.05 if it is the same subscript for the same sub-variable

Significance at significance level of p < 0.005

### Prevalence of S. aureus, resistance, MRSA and socio-demographic factors

Table 1 shows the distribution of the socio-demographic factors within the *S. aureus* carrier group, the group of carriers with AR, the group of carriers with AR besides PEN, and the MRSA group. Males, younger adults and those living in the countryside showed a statistically significant increased likelihood to be *S. aureus* carriers. Additionally, Table 1 shows that 64.8 % of the *S. aureus* carriers had resistances aside from PEN. These cases were more frequent in persons living in rural areas.

The number of MRSA carriers was too small to detect serious differences in socio-demographics. Also for *S. aureus* carriers with resistance to one or more antibiotics, no differences in the socio-demographics could be found.

In the first adjusted multivariable logistic regression model, positive associations with *S. aureus* carrier status could be found for those of the male sex, between the age of 19 and 39 years, living in an rural area, and working in the health care sector (Table 2). Additionally, in the third adjusted model living in a rural area was found to increase the probability of being a *S. aureus* carrier with resistances besides PEN (Table 2).

**Table 2:** Regression model for *S. aureus* carriers, carriers with resistances, and carriers with resistances besides PEN adjusted for GP practices

		*S. aureus* carrier		*S. aureus* carrier with resistances		*S. aureus* carrier with resistances besides PEN	
Variable	Subvariable	OR (95% CI)	*p*-value	OR (95% CI)	*p*-value	OR (95% CI)	*p*-value
Sex	Female	1.0		1.0		1.0	
	Male	1.60 (1.32–1.95)	<0.001	0.81 (0.56–1.19)	0.283	0.93 (0.64–1.35)	0.709
Age	4–18	1.54 (0.86–2.75)	0.148	1.17 (0.43–3.16)	0.764	0.61 (0.21–1.78)	0.365
	19–39	1.34 (1.02–1.77)	0.038	1.36 (0.78–2.35)	0.276	1.58 (0.91–2.74)	0.105
	40–64	1.17 (0.90–1.51)	0.235	1.08 (0.65–1.77)	0.773	1.06 (0.65–1.73)	0.820
	65+	1.0		1.0		1.0	
Location of residence	Urban	1.0		1.0		1.0	
	Rural	1.37 (1.12–1.67)	0.002	1.27 (0.86–1.90)	0.232	1.53 (1.02–2.30)	0.041
Job	Healthcare	1.47 (1.02–2.13)	0.041	0.94 (0.46–1.92)	0.857	0.84 (0.42–1.67)	0.615
	Livestock farming	0.79 (0.45–1.40)	0.418	0.65 (0.23–1.86)	0.423	0.54 (0.19–1.55)	0.250
	Kindergarten teacher/nanny	0.63 (0.27–1.50)	0.297	2.13 (0.25–18.20)	0.489	2.63 (0.29–23.68)	0.389
	Not known	0.71 (0.48–1.03)	0.067	0.95 (0.47–1.94)	0.895	0.80 (0.39–1.65)	0.545
	Others	1.0		1.0		1.0	
Nagelkerkes R^2^		0.050		0.074		0.066	

### Multidrug-resistance of *S. aureus* and associations with socio-demographic factors

If *S. aureus* strains were resistant to three or more antibiotics, nearly all were resistant to PEN, AZITH, ERY, and CLIND (Table 3). *S. aureus* strains that were resistant against PEN most commonly demonstrated isolated resistant against PEN, but did show secondary resistances against three other AB, mainly AZITH, ERY and CLIND (Table 4). Out of the MRSA strains, five were resistant against other AB besides OXA and PEN (Table 5). Of all *S. aureus* carriers, with the exception of MRSA carriers, 13.7 % (n = 72) were identified as multi-drug resistant strains. Persons living in urban areas were found to have significantly more frequent multi-drug resistant strains (Table 6).

**Table 3:** Number of resistances of *S. aureus* against AB (except MRSA) (n = 541)

Nr. of res.	Percent (n)	AB resistance ranked by frequency in percent (n)
0	30.3 (164)	-
1	53.3 (288)	97.6 (281) PEN, 1.4 (4) TETRA, 0.7 (2) GENTA, 0.3 (1) CIP
2	3.3 (18)	83.3 (15) PEN, 44.4 (8) TETRA, 38.9 (7) GENTA, 22.2 (4) CIP, 5.6 (1) AZITH, 55.6 (1) ERY
3	4.6 (25)	96.0 (24) AZITH, 92.0 (23) ERY, 64.0 (16) CLIND, 32.0 (8) PEN, 8.0 (2) CIP, 3.8 (1) GENTA, 4.0 (1) TETRA, 4.0 (1) TRISUL
4	8.1 (44)	100.0 (44) ERY, 100.0 (44) AZITH, 95.5 (42) PEN, 95.5 (42) CLIND, 4.5 (2) GENTA, 4.5 (2) TETRA
5	0.4 (2)	100.0 (2) ERY, 100.0 (2) AZITH, 100.0 (2) TETRA, 100.0 (2) PEN, 100.0 (2) CLIND

**Table 4:** Number of resistances of *S. aureus* against AB besides PEN (n = 356)

Nr. of res.	Percent (n)	AB resistance ranked by frequency in percent (n)
	79.0 (281)	PEN 100.0 (281)
1	5.0 (18)	+ CIP 22.2 (4), GENTA 27.8 (5), OXA 16.7 (3), TETRA 33.3 (6)
2	2.5 (9)	+ AZITH 77.8 (7), CIP 11.1 (1), ERY 77.8 (7), GENTA 11.1 (1), OXA 11.1 (1), TETRA 11.1 (1)
3	12.0 (43)	+ AZITH 100.0 (43), CLIND 95.3 (41), ERY 100.0 (43), GENTA 2.3 (1), OXA 2.3 (1)
4	0.8 (3)	+ AZITH 100.0 (3), CLIND 66.7 (2), ERY 100.0 (3), OXA 33.3 (1), TETRA 100.0 (3)
5	0.6 (2)	+ AZITH 100.0 (2), CIP 50.0 (1), CLIND 100.0 (2), ERY 100.0 (2), OXA 100.0 (2), TETRA 50.0 (1)

**Table 5:** Number of resistances of MRSA strains in addition to PEN and OXA (n = 8)

Nr. of res.	Percent (n)	AB resistance ranked by frequency in percent (n)
	37.5 (3)	OXA & PEN 100.0 (3)
1	12.5 (1)	+ GENTA 100.0 (1)
2	12.5 (1)	+ AZITH 100.0 (1) + ERY 100.00 (1)
3	12.5 (1)	+ AZITH 100.0 (1) + ERY 100.0 (1) + TETRA 100.0 (1)
4	25.0 (2)	+ AZITH 100.0 (2) + CIP 50.0 (1) + CLIND 100.0 (2) + ERY 100.0 (2) + TETRA 50.0 (1)

**Table 6:** Distribution of the socio-demographic variables in relation to the number of antibiotics resistant to *S. aureus* (except MRSA)

Variable	Sub-variable	Number of antibiotics resistant to *S. aureus*				*p*-value
		0	1	2	3+	
		% (n)	% (n)	% (n)	% (n)	
Sex	Female	27.8 (70)	52.4 (132)	4.8 (12)	15.1 (38)	0.175
	Male	31.8 (91)	54.5 (156)	2.1 (6)	11.5 (33)	
Age	4–18	28.6 (8)	46.4 (13)	0	25.0 (7)	
	19–39	26.5 (43)	61.7 (100)	2.5 (4)	9.3 (15)	0.224
	40–64	31.9 (79)	50.0 (124)	3.6 (9)	14.5 (36)	
	65+	33.0 (34)	50.5 (52)	4.9 (5)	11.7 (12)	
Educational level	Primary	30.6 (81)	56.6 (150)	3.8 (10)	9.1 (24)	
	Secondary	31.4 (60)	51.3 (98)	1.6 (3)	15.7 (30)	0.127
	Tertiary	31.4 (22)	45.7 (32)	5.7 (4)	17.1 (12)	
Country of origin	Austria	31.5 (145)	52.7 (243)	2.8 (13)	13.0 (60)	
	EU 15+	22.7 (5)	54.5 (12)	4.5 (1)	18.2 (4)	0.748
	New EU 28	25.0 (4)	56.3 (9)	6.3 (1)	12.5 (2)	
	Others	25.0 (9)	58.3 (21)	8.3 (3)	8.3 (3)	
Location of residence	Urban	33.3 (66)	44.9 (89)_a_	4.0 (8)	17.7 (35)_a_	0.014
	Rural	28.5 (98)	58.1 (200)_b_	2.9 (10)	10.5 (36)_b_	
Job	Health care	30.8 (12)	46.2 (18)	5.1 (2)	17.9 (7)	
	Livestock farming	37.5 (6)	43.8 (7)	6.3 (1)	12.5 (2)	
	Kindergarten teacher/nanny	14.3 (1)	57.1 (4)	0	28.6 (2)	0.926
	Others	29.9 (128)	54.9 (235)	3.0 (13)	12.1 (52)	
	Not known	32.7 (17)	48.1 (25)	3.8 (2)	15.4 (8)	

^a,b^The subscript letters represent a subset of the variable category which is not significantly different at a significance level of p < 0.05 if it is the same subscript for the same sub-variable

Significance at significance level of p < 0.005

## Discussion

The present study evaluated the prevalence and resistance patterns of commensal nasal *S. aureus* strains from urban and rural GP patients in Austria. The results subsequently show differences associated with socio-demographic factors in these populations.

Males, young adults, persons living in the countryside and those persons working in the healthcare sector were significantly more likely to be *S. aureus* carriers. Rural areas demonstrated a clear association with *S. aureus* carrier status, even when adjusting for GP practice. This is likely a consequence of members of rural communities having more contact with *S. aureus*. Rural areas include those with farmland, which may suggest a higher exposure to the influence of livestock breeding. This has previously been described in literature as a risk factor for *S. aureus* carrier and resistance carrier status [12–15]. However, interestingly, livestock workers did not demonstrate a higher prevalence of carrier status in our analysis, though this could be due to the small sample size of this subgroup. It was additionally recognized that males are more likely to be *S. aureus* carriers, with an OR of 1.60 compared to women. This result is supported by other studies that have also observed higher *S. aureus* carriage in men [22]. Possible reasons for this finding include host genetics or human innate immune factors, smoking status, or vitamin D levels [39–41].

Compared to other professions, healthcare workers were found to have a greater likelihood to be *S. aureus* carriers (OR of 1.47). This finding is not surprising, given the regular exposure and proximity of healthcare workers to variable and resistant strains of microbials in hospital and ambulatory settings. However, it does offer important implications for healthcare workers and the health care system. In the consultation setting, healthcare workers should be considered to have a higher likelihood to carry *S. aureus* strains and an increased chance to transfer these to their patients. Therefore, hand-hygiene following patient contact is essential to hinder the transfer [42].

Nearly 30 % of *S. aureus* strains identified were susceptible to all antibiotics tested. The vast majority (64.8 %) of commensal *S. aureus* strains was resistant to small-spectrum PEN, followed by the macrolide antibiotics AZITH and ERY as well as CLIND, with a resistance rate around 12 %. Comparing these findings to those in other European countries, it becomes clear that Austria is nearly average in terms of prevalence rates when compared to other European nations [8]. As it is recognized that one of the corner stones for the containment of AR is the appropriate prescription of antibiotics in the outpatient sector [28], this finding could support to the avoidance of prescription of broad-spectrum antibiotics to treat *S. aureus* infections in the community. This is because of low levels of resistance in these settings, aside from PEN resistances in ambulatory populations.

With regard to the overall resistances of the *S. aureus* strains carried, no associations with socio-demographics were directly identified. However, for the *S. aureus* strains that were resistant to antibiotics besides PEN, associations with living in rural areas could be found. This goes in line with the *S. aureus* carriers that were more frequently found in rural areas. In contrast, multi-drug resistances were observed more often in individuals living in urban areas. The higher rates of multi-drug resistant *S. aureus* strains in individuals living in urban areas could be a consequence of higher travel activity compared to those living in rural areas [11]. It could additionally be surmised that persons living in urban areas have easier access to ambulatory or hospital sectors, and thus have a higher likelihood to present for even simple conditions, such as a common cold. Due to the fact that Austria has no gatekeeping system in primary care, patients may access care at any point and as often as they wish. Thus, these patients could have a higher probability of being exposed to antibiotics [43]. In line with this, Hoffmann et al. found that in Eastern Austria, approximately one third of persons that do not get an antibiotic prescription from the GP will subsequently present to a specialist to receive one [44].

In this study with participants without infectious diseases, we observed low rates of MRSA in Austria, with only 1.5 % of *S. aureus* carriers having MRSA. An important finding of our study is that there exists a difference among the MRSA rates previously examined in hospital samples and those samples from sick patients presenting to the ambulatory sector with a bacterial infectious disease. For example, in Austria in 2013, the Austrian resistance report stated that hospitalized patients were found to have invasive (blood and cerebrospinal fluid) MRSA strains with a prevalence of 9.1 %, and non-invasive MRSA strains in 6.7 % of cases studied. Meanwhile, non-invasive MRSA strains were detected from sick persons in the outpatient sector with a prevalence of 4.0 % [21].

If *S. aureus* strains were resistant to three or more antibiotics, we observed that nearly all of these were resistant to PEN, AZITH, ERY, and CLIND (Table 3, Table 4 and Table 5). This has profound and practical implication for care of patients in Austria, particularly as prescribers can look to utilize other antibiotics in patient populations with high likelihood for multiple resistances.

### Strength and limitations

A strength of the present study was the community setting in which patients were drawn from a GP population who visited the practice for a non-infectious reason. This is particularly important as no previous study of depth has been carried out in this population. Another strength was the large sample size and the similarity of the sample with the Austrian population with regard to sex, age and educational level [45]. However, as the study population has been recruited in general practices we lacked participants among those that otherwise did not present to a GP within this time frame. Other limitations were the non-randomized recruitment strategy of GPs and patients and the fact that the questionnaire was available exclusively in German. It may be speculated that more GPs and patients interested in the topic of AR participated in the study, which would have led to an underestimation of the real resistance pattern of antibiotics, especially, regarding non-German speaking migrants. Finally, socio-demographic factors explain only about 5 % of variance of the *S. aureus* prevalence and resistance rates, suggesting that other factors such as host genetics or human innate immune factors, smoking status, vitamin D levels, travel activities, or exposure to antibiotics may play a role as well. As this study is cross-sectional there is limitation to the explanatory power.

## Conclusion

Emergence of increasingly resistant strains of microbials has been recognized by the WHO and others as a global health threat. AR is heavily influenced by dynamics that occur outside of hospital settings, such as prescribing patterns and livestock breeding. Despite this, the majority of studies of AR in Europe have occurred in hospitalized patients or sick ambulatory patients presenting with bacterial infections. This study, in contrast, describes the prevalence and resistance pattern of commensal *S. aureus* in community-dwelling GP patients in Austria and shows differences between socio-demographic groups. Particularly, differences were identified in the prevalence and multi-drug resistances of commensal *S. aureus* between the sexes and individuals living in rural and urban areas in Austria. More than two thirds of all *S. aureus* carriers had resistant strains, though the vast majority was resistant against small-spectrum PEN only. Physicians should consider these findings when determining therapeutic options with antibiotics. Particularly, this finding could lend support to the avoidance of prescription of broad-spectrum antibiotics to treat *S. aureus* infections in rural community settings.

As we continue to witness a global rise in AR, it is imperative that acknowledged patterns of resistance are accurate and reflect true prevalence at the community level. Responding to outbreaks in resistant strains will require an understanding of social and demographic factors as well. We urge the further study of communities and, otherwise healthy, populations in order to continue to elucidate representative prevalence and resistance patterns. Ideally, continual surveillance of resistance patterns and antibiotic consumption in the outpatient setting should be carried out to detect changes early and to inform evidence-based decisions in a timely manner.

## Abbreviations

APRES study: Appropriateness of Prescribing Antibiotics in Primary Health Care in Europe study
AR: Antibiotic resistance
AZITH: Azithromycin
CIP: Ciprofloxacin
CLIND: Clindamycin
DAPT: Daptomycin
EFTA: European Free Trade Association
ERY: Erythromycin
EU: European Union
EUCAST: European Committee on Antimicrobial Susceptibility Testing
GENTA: Gentamicin
GP: General practitioner
LIN: Linezolid
MRSA: Methicillin-resistant *Staphylococcus aureus*
OR: Odds ratio
OXA: Oxacillin
PEN: Penicillin
*S. aureus*: *Staphylococcus aureus*
STROBE: International, collaborative initiative of epidemiologists, methodologists, statisticians, researchers and journal editors involved in the conduct and dissemination of observational studies
TETRA: Tetracycline
TRISUL: Trimethoprim-sulfamethoxazole
VANC: Vancomycin
WHO: World Health Organization
